# Vasodilatory Peripheral Response and Pain Levels following Radiofrequency Stressor Application in Women with Fibromyalgia

**DOI:** 10.3390/biomedicines12010142

**Published:** 2024-01-10

**Authors:** Antonio Casas-Barragán, Alba Muñoz-Revilla, Rosa María Tapia-Haro, Francisco Molina, María Correa-Rodríguez, María Encarnación Aguilar-Ferrándiz

**Affiliations:** 1 Department of Physical Therapy, Faculty of Health Sciences, University of Granada (UGR), 18016 Granada, Spain; antoniocb@ugr.es (A.C.-B.); rtapia@ugr.es (R.M.T.-H.); fjmolina@ugr.es (F.M.); e_aguilar@ugr.es (M.E.A.-F.); 2 Instituto de Investigación Biosanitaria ibs.GRANADA, 18012 Granada, Spain; 3 Biomedicine Program, Department of Physical Therapy, Faculty of Health Sciences, University of Granada (UGR), 18071 Granada, Spain; albamure@correo.ugr.es; 4 Department of Nursing, Faculty of Health Sciences, University of Granada (UGR), Ave. de la Ilustración, 60, 18016 Granada, Spain

**Keywords:** fibromyalgia, radiofrequency, peripheral temperature, core body temperature, thermography, pain levels

## Abstract

Fibromyalgia (FM) is a syndrome of unknown pathogenesis that presents, among other symptoms, chronic widespread musculoskeletal pain. This study aims to analyze the effects of radiofrequency on core body temperature and the peripheral temperature of the dorsal surfaces and palms of the hands and its association with pain levels in patients with FM. A case-control observational study was conducted with a total of twenty-nine women diagnosed with FM and seventeen healthy women. Capacitive monopolar radiofrequency was applied to the palms of the hands using the Biotronic Advance Develops device. Peripheral hand temperature was analyzed using a thermographic camera, and core body temperature was analyzed with an infrared scanner. Pressure pain thresholds (PPTs) and electrical pain were recorded with an algometer and a Pain Matcher device, respectively. A significant decrease was observed in women with FM in pain electrical threshold (95% CI [0.01–3.56], p = 0.049), electrical pain (95% CI [2.87–10.43], *p* = 0.002), dominant supraspinatus PPT (95% CI [0.04–0.52], *p* = 0.023), non-dominant supraspinatus PPT (95% CI [0.03–0.60], *p* = 0.029), and non-dominant tibial PPT (95% CI [0.05–0.89], *p* = 0.031). Women with FM have increased hypersensitivity to pain as well as increased peripheral temperature after exposure to a thermal stimulus, such as radiofrequency, which could indicate disorders of their neurovascular response.

## 1. Introduction

Fibromyalgia (FM) is a disorder characterized by the presence of chronic diffuse musculoskeletal pain accompanied by a series of other neuropathic, somatic, and psychological symptoms [1,2]. The prevalence of FM is estimated to be 1.78% worldwide, and it is highest in Europe, where the figure rises to 2.64%. In terms of gender, FM affects many more women (4.49%) than men (0.29%), at a ratio of 15 to 1 [2].

The pathogenesis of FM remains unknown, although it has been recognized that the principal factor is related to central nervous system (CNS) alterations [3,4]. However, recently, other authors have proposed new hypotheses that point to the presence of blood microcirculation alterations generated by skin disorders at the peripheral level and changes in the innervation of the arteriovenous anastomoses (AVAs) in the palms of the hands as causative factors for FM [5,6]. AVAs are small blood vessels that are innervated by the sympathetic system and adrenergic fibres and play an important role in the body’s thermoregulatory function by keeping the body’s temperature in the thermoneutral zone [7]. In this regard, Albrecht et al. [5] found an excess of peptidergic sensory innervation over the noradrenergic sympathetic function of AVAs, resulting in peptidergic fibre stimulation, activating the local “axon reflex”, and triggering the release of substance P and calcitonin gene-related peptide (CGRP) into the bloodstream [5]. These two neuropeptides are potent vasodilators that contribute to neurogenic inflammation processes, thus generating vascular changes at the peripheral level and potentially affecting sweating and heat-generation mechanisms, as well as, among other things, chronic pain processes, such as peripheral sensitization [8]. This points to the existence of a pathology of small-caliber nerve fibres leading to an altered neurovascular response with structural, functional, and sensory changes, thus supporting the idea that possible vasculopathy can partially explain the perfusion deficits and their relationship with FM symptoms [9,10]. Given all of this, the involvement of the AVAs would lead to impaired temperature regulation, resulting in a lack of blood circulation, nutrition, and oxygenation at the deep musculoskeletal tissue level [11,12].

Additionally, non-percutaneous pulsed radiofrequency (PRF) is an electrotherapy modality that has been used to treat pain in various chronic pain populations [13,14]. Due to the impedance of the tissue to generate heat instead of transferring it directly, this type of current could act by producing vascular changes in the local area, thus promoting vasodilation in the region of the arterioles and facilitating vasoconstriction by neural reflex [14]. Although there are studies that suggest a relationship between temperature modulation after exposure to a cold stressor and the intensity of pain and other symptoms in patients with FM [15,16], there are no scientific studies evaluating the vascular–thermal component and its relationship to symptomatic changes after the application of a heat stressor specifically induced by PRF.

Against this background, we hypothesized that the application of a thermal heat stimulus would generate changes at the vascular level that would affect the perception of painful stimuli in women with FM. For this reason, the purpose of this study was to analyze the immediate effects on core body temperature and peripheral hand surface temperature, as estimators of vascular reactivity, following the application of a vasodilator radiofrequency protocol and to examine the association of these results with pain intensity, electrical pain threshold, and pressure pain threshold (PPT) in a population of women diagnosed with FM as well as healthy controls.

## 2. Materials and Methods

### 2.1. Design and Participants

An observational case-control study was conducted between May and October 2022. A total of 29 women with FM from two associations, the Granada Fibromyalgia Association (AGRAFIM) and the Jaén Fibromyalgia Association (AFIXA), and 17 healthy controls matched by age and sex were recruited. The participants were duly informed about the assessment tools and signed the corresponding informed consent. This study was approved by the University Hospital of Granada Research Ethics Committee (Granada, Spain) with approval number 1718-N-18 in accordance with the ethical criteria established in the Declaration of Helsinki (modified in 2013) of the World Medical Association (WMA) for the conduct of research projects.

The inclusion criteria for both groups were (1) female sex; (2) between 18 and 70 years of age; (3) diagnosis of FM (FM group) by a Spanish National Health Service rheumatologist in accordance with the criteria of the American College of Rheumatology (ACR); (4) no previous rheumatic disease; and (5) no regular physical activity. The exclusion criteria for both groups were: (1) presence of cardiac, renal, or hepatic insufficiency; (2) severe physical disability; (3) infectious processes, fever, hypertension/hypotension, or respiratory alterations; (4) severe psychiatric disease; (5) previous surgery; (6) non-compliance with the prescribed pharmacological regimen; (7) skin alterations; (8) presence of associated comorbidities (chemical hypersensitivity syndrome, chronic fatigue syndrome, interstitial cystitis, etc.); (9) treatment with beta blockers, anticonvulsant therapy, steroid therapy, or disease-modifying drugs in the 4 weeks prior to this study; (10) regular use of immunosuppressive drugs; and (11) pregnancy or lactation.

### 2.2. Definition of Variables

When the participants were accepted into this study, their standardized medical history and sociodemographic data were collected by a physiotherapist. During a second visit, we conducted a clinical and instrumental assessment. At this point, the patients were instructed not to exert themselves physically and to avoid caffeine, alcohol, and nicotine.

#### 2.2.1. Peripheral Vascular Assessment and Central Thermoregulation

The baseline pattern of the vascular component of the hands was analyzed using an infrared thermography (IRT). IRT provides indirect information on blood flow, circulation, thermal properties, and the thermoregulatory functionality of the skin tissue [17]. The process of how the thermographic evaluations were conducted on the dorsal surfaces and palms of both hands has been described in previous studies [18].

On the other hand, a pocket infrared thermometer (Infrared Dermal Thermometers DT-1001-LN, Exergen Corporation, Watertown, MA, USA) was used to measure the core body temperature by recording the temperature at the axillary and external auditory canal levels [19].

#### 2.2.2. Radiofrequency Vasodilation

**Figure 1:**
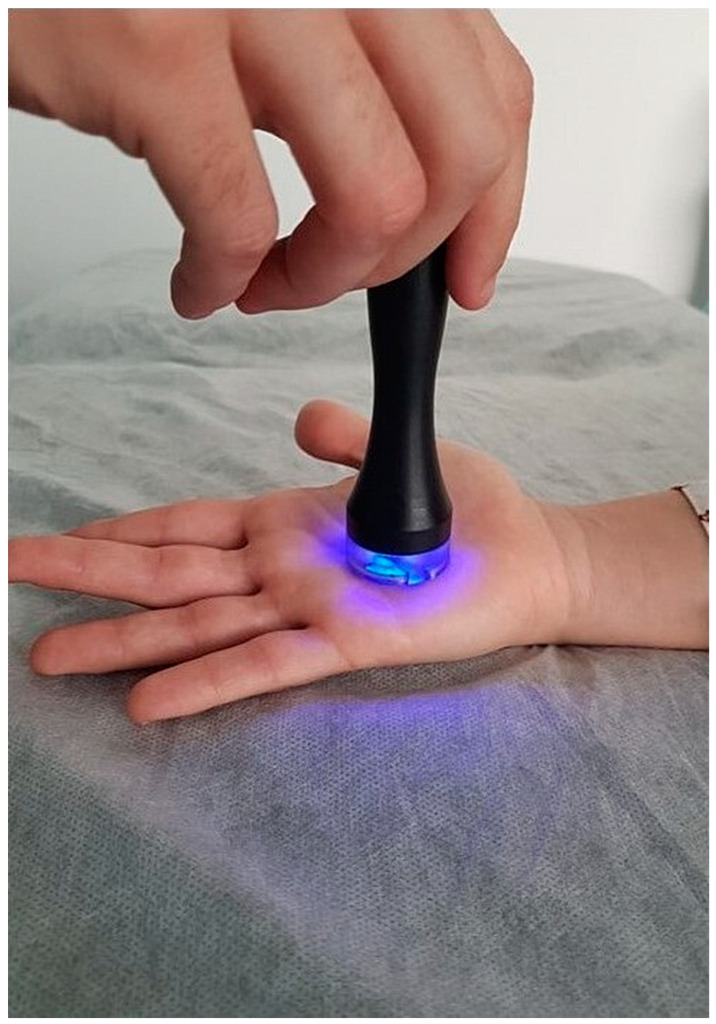
Application of pulsed radiofrequency on the palmar centre region of the hand of a woman with fibromyalgia.

Devices that apply radiofrequency cause the cellular structures in its path to oscillate, thus resulting in increased intermolecular motion. As the current flows alternately, molecular collisions increase, thereby creating thermal energy (heat) [14]. In accordance with a previous study, a model Xcultp^®^ (Biotronic Advance Develops S.L., Granada, Spain) was used to provide a monopolar dielectric capacitive radiofrequency with a wave frequency of 850 kHz, digitally modulated up to 160 Hz, and with a carrier wave amplitude of 8 Kv [20]. All subjects were placed in a seated position with the forearms supinated and resting on a stretcher, where the emission of radiofrequency was applied using a 5 cm^2^ circular head for a total of 10 min on the palm of each hand through an almond oil conductor. Figure 1 shows the application of PRF at the palmar centre of the hands.

#### 2.2.3. Subjective Perception of Pain and Determination of Pressure Pain Thresholds

To assess the patients’ subjective pain intensity, the visual analogue scale (VAS) was used; on a 100 mm long line, this runs from 0 points (no pain) to 10 points (maximum pain imaginable) [21]. This scale has been demonstrated to have good sensitivity and specificity for patients with FM [22].

Pressure pain thresholds (PPTs) were recorded using a digital pressure algometer (Wagner Instruments, Greenwich, CT, USA) at the 9 points established by the ACR. Bilaterally, three measurements were taken at each point with a 30 s rest period in between, so that the final variable was the arithmetic mean of the three assessments. The reliability for this method of measuring the PPT has been found to be high [23].

#### 2.2.4. Assessment of Pain in Response to Electrical Stimuli

Pain electric threshold and pain electrical intensity were measured using a Pain Matcher device (Cefar-Compex Scandinavia Inc., Medical AB, Lund, Sweden). The participants were instructed to grip the Pain Matcher (with the electrical stimulation unit) firmly between the right thumb and forefinger in order to record electrical pain threshold and pain magnitude. The Pain Matcher has been shown to have good reliability in a number of studies [24,25,26].

### 2.3. Statistical Analysis

G*Power Version 3.1.9.7 software (Heinrich Heine University Düsseldorf, Germany) was used to estimate the sample size. Taking into account previous results regarding general pain in FM patients through monopolar radiofrequency [20], it is necessary to include a minimum of 15 subjects per group to provide a power of 80% and an alpha level (α) of 0.05.

The data were analyzed using SPSS version 24.0 for Windows (IBM Corporation, Armonk, NY, USA). The Kolmogorov–Smirnov test (*p* > 0.05) was used to check the normality of the variables. To compare mean differences between the FM and healthy groups of women, the unpaired Student’s *t*-test with a 95% confidence interval (95% CI; value α = 0.05) for continuous data and the chi-square test (χ^2^) for categorical variables were used. We performed a 2 × 2 mixed model repeated-measures analysis of variance (ANOVA) to test the effect of PRF on temperature as the primary measure and pain levels as secondary measures, with time (pre and post radiofrequency) being the within-subject variable and group (FM or control) as the between-subjects variable. The effect size was calculated according to Cohen’s d. A value of <0.2 indicated a negligible effect size, a value between ≥0.2 and <0.5 indicated a small effect size, a value between ≥0.5 and <0.8 indicated a moderate effect size, and a value ≥0.8 indicated a large effect size.

## 3. Results

### 3.1. Demographic and Clinical Data

The sociodemographic characteristics of the participants in each group are shown in Table 1. No statistically significant differences were found between the FM group and healthy women for any of the established variables.

### 3.2. Differences in Pain Levels following Thermal Heat Stimulus

**Figure 2:**
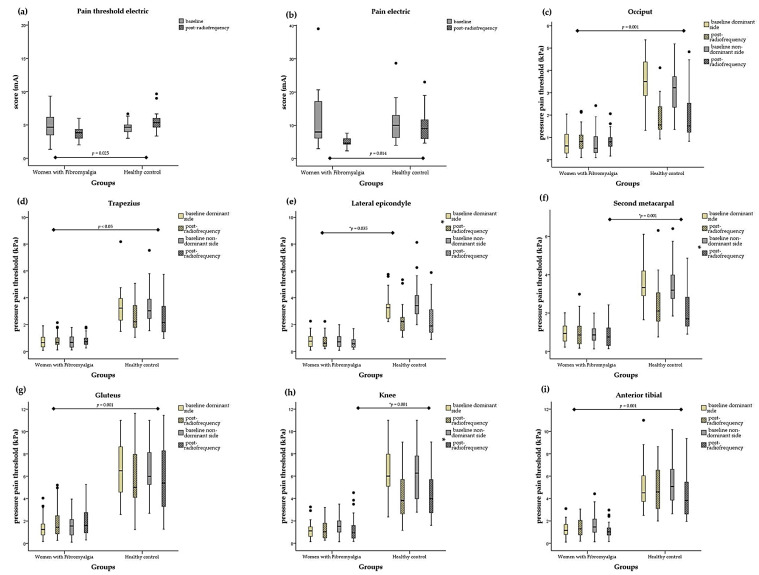
Box plots of pain levels after radiofrequency treatment for the women with fibromyalgia and healthy controls. The box plots show the comparison of baseline and post-radiofrequency data from (**a**) pain threshold electric, (**b**) pain electric, (**c**) occiput pressure pain threshold (PPT), (**d**) trapezius PPT, (**e**) epicondyle PPT, (**f**) second metacarpal PPT, (**g**) gluteus PPT, (**h**) knee PPT, and (**i**) anterior tibial PPT between the women with fibromyalgia and healthy controls. In the box plots, the boundary of the box closest to 0 indicates the 25th percentile, the black line within the box marks the median, and the boundary of the box farthest from 0 indicates the 75th percentile. Whiskers above and below the box indicate the 10th and 90th percentiles. * Significant group, *p* < 0.05. Note that black dots indicate the extreme values.

The effect of treatment with radiofrequency and final values regarding pain levels in each group are shown in Table S1. The ANOVA analysis revealed significantly lower electrical threshold (F = 5.51, *p* = 0.025) and electrical pain (F = 6.75, *p* = 0.014) values in the FM group. Intra-group analysis also revealed a significant decrease in the FM group for the electrical pain threshold score (*p* = 0.049) and in the electrical pain intensity score (*p* = 0.002). Likewise, we observed significant differences between the FM group and healthy women in the occiput (dominant: F = 33.37, *p* = 0.001; non-dominant: F = 13.15, *p* = 0.001), trapezius (dominant: F = 18.79, *p* = 0.001; non-dominant: F = 7.80, *p* = 0.008), epicondyle dominant (F = 4.74, *p* = 0.035), gluteus (dominant: F = 54.07, *p* = 0.001; non-dominant: F = 58.99, *p* = 0.001), knee non-dominant (F = 43.09, *p* = 0.001), second metacarpal non-dominant (F = 12.29, *p* = 0.001), and anterior tibial PPTs (dominant: F = 16.23, *p* = 0.001; non-dominant: F = 23.44, *p* = 0.001). In the intra-group analysis, a significant decrease in PPTs in the control group was observed in the occiput dominant (*p* = 0.036), epicondyle (dominant: *p* = 0.011; non-dominant: *p* = 0.008), knee (dominant: *p* = 0.008; non-dominant: *p* = 0.035), and second metacarpal (dominant: *p* = 0.006; non-dominant: *p* = 0.003). For the women diagnosed with FM, there was a significant decrease in the supraspinatus (dominant: *p* = 0.023; non-dominant: *p* = 0.029) and the anterior tibial non-dominant (*p* = 0.031) and a significant increase in the gluteus dominant PPTs (*p* = 0.016). Figure 2 shows the significant changes in pain variables following radiofrequency stressor application in women with fibromyalgia and healthy controls.

### 3.3. Differences in Vascular Response following Thermal Heat Stimulus

Tables S2 and S3 show the changes in core body temperature and peripheral temperature of the hands after the radiofrequency intervention. The results revealed significant differences in tympanic temperature (F = 12.39, *p* = 0.001) and axillary temperature (F = 6.84, *p* = 0.012) between the FM group and healthy women. In the intra-group analysis, pre- and post-radiofrequency differences were observed, with a significant decrease in the control group in terms of both core body temperature (*p* = 0.009) and axillary temperature (*p* = 0.014).

**Figure 3:**
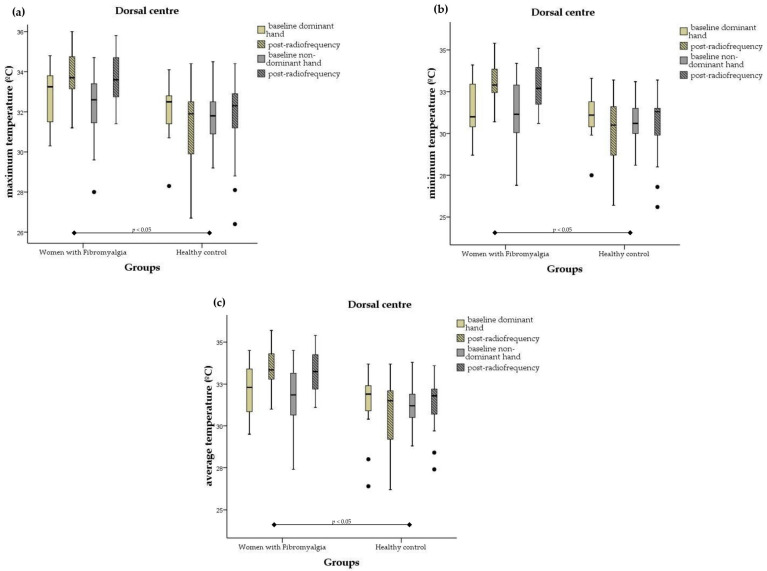
Box plots of peripheral temperature values at dorsal sites of the hands after radiofrequency treatment for the women with fibromyalgia and healthy controls. The box plots show the comparison of baseline and post-radiofrequency data from (**a**) dorsal centre maximum temperature, (**b**) dorsal centre minimum temperature, and (**c**) dorsal centre mean temperature. In the box plots, the boundary of the box closest to 0 indicates the 25th percentile, the black line within the box marks the median, and the boundary of the box farthest from 0 indicates the 75th percentile. Whiskers above and below the box indicate the 10th and 90th percentiles. Note that black dots indicate the extreme values.

On the other hand, the peripheral temperature of the dorsal surface of the hands in the women with FM was significantly higher in the dorsal centre (maximum dominant side: F = 7.76, *p* = 0.008; maximum non-dominant side: F = 4.93, *p* = 0.032; minimum dominant side: F = 12.65, *p* = 0.001; minimum non-dominant side: F = 8.47, *p* = 0.006; mean dominant side: F = 8.96, *p* = 0.005; mean non-dominant side: F = 6.82, *p* = 0.012). Figure 3 shows the significant changes in the peripheral temperature of the skin at the dorsal surfaces of the hands following radiofrequency stressor application in women with fibromyalgia and healthy controls.

**Figure 4:**
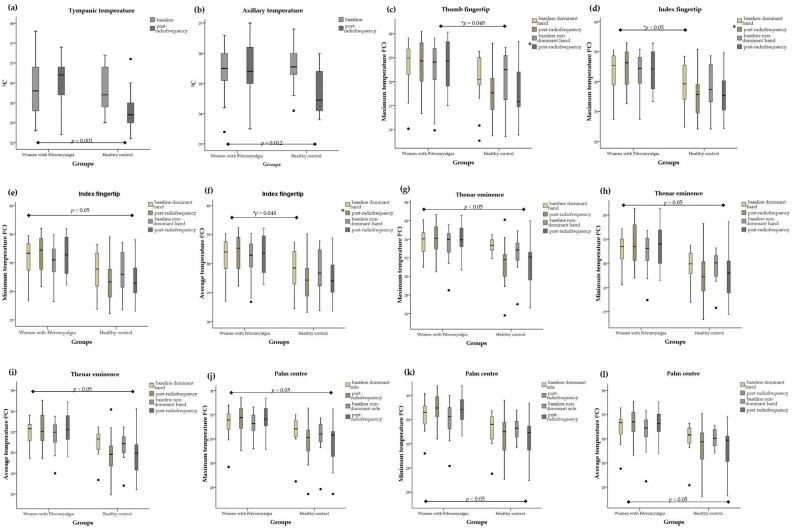
Box plots of core body temperature and peripheral temperature values at palm sites of the hands after radiofrequency treatment for the women with fibromyalgia and healthy controls. The box plots show the comparison of baseline and post-radiofrequency data from (**a**) tympanic temperature, (**b**) axillary temperature, (**c**) thumb fingertip maximum temperature, (**d**) index fingertip maximum temperature, (**e**) index fingertip minimum temperature, (**f**) index fingertip mean temperature, (**g**) thenar eminence maximum temperature, (**h**) thenar eminence minimum temperature, (**i**) thenar eminence mean temperature, (**j**) palm centre maximum temperature, (**k**) palm centre minimum temperature, and (**l**) palm centre mean temperature. In the box plots, the boundary of the box closest to 0 indicates the 25th percentile, the black line within the box marks the median, and the boundary of the box farthest from 0 indicates the 75th percentile. Whiskers above and below the box indicate the 10th and 90th percentiles. * Significant group, *p* < 0.05. Note that black dots indicate the extreme values.

At the palmar sites of the hands, the peripheral temperature was significantly lower in the control group on the maximum thumb fingertip non-dominant side (F = 2.09, *p* = 0.049), index fingertip (maximum dominant side: F = 4.06, *p* = 0.050; minimum dominant side: F = 4.37, *p* = 0.043; minimum non-dominant side: F = 4.15, *p* = 0.048; mean dominant side: F = 4.14, *p* = 0.048), and thenar eminence (maximum dominant side: F = 11.32, *p* = 0.002; maximum non-dominant side: F = 6.70, *p* = 0.013; minimum dominant side: F = 6.01, *p* = 0.018; minimum non-dominant side: F = 4.47, *p* = 0.041; mean dominant side: F = 8.91, *p* = 0.005; mean non-dominant side: F = 7.02, *p* = 0.011). For the FM group, the peripheral temperature of the palms of the hands was significantly higher in the palm centre (maximum dominant side: F = 7.11, *p* = 0.011; maximum non-dominant side: F = 5.10, *p* = 0.029; minimum dominant side: F = 8.03, *p* = 0.007; minimum non-dominant side: F = 8.71, *p* = 0.005; mean dominant side: F = 7.41, *p* = 0.009; mean non-dominant side: F = 6.92, *p* = 0.012). Figure 4 shows the significant changes in core body temperature and peripheral temperature of the skin at the palms of the hands following radiofrequency stressor application in women with fibromyalgia and healthy controls.

**Figure 5:**
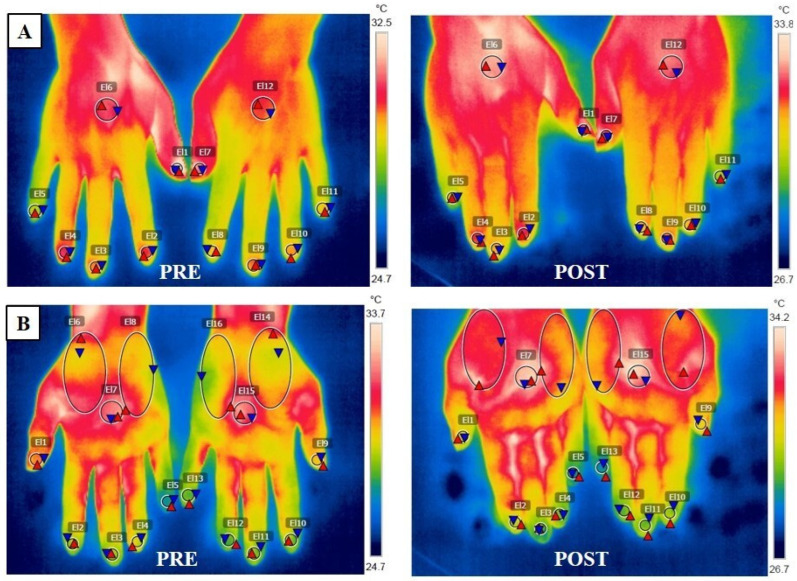
Pre- and post-thermography images of the hands following radiofrequency of a woman diagnosed with fibromyalgia (**A**) Image of the dorsal thermography of the hands (El1 and El7 = thumb finger; El2 and El8 = index finger; El3 and El9 = middle finger; El4 and El10 = ring finger; El5 and El11 = pinkie finger; El6 and El12 = dorsal centre). (**B**) Image of the palmar thermography of the hands (El1 and El9 = thumb finger; El2 and El10 = index finger; El3 and El11 = middle finger; El4 and El12 = ring finger; El5 and El13 = pinkie finger; El6 and El14 = thenar eminence; El7 and El15 = palmar centre; El8 and El16 = hipothenar eminence).

Finally, intra-group analysis showed a significant increase in the temperature at the dorsal centre (maximum dominant side: *p* = 0.002; maximum non-dominant side: *p* = 0.001; minimum dominant side: *p* = 0.001; minimum non-dominant side: *p* = 0.001; mean dominant side: *p* = 0.001; mean non-dominant side: *p* = 0.001), at the palm centre (minimum dominant side: *p* = 0.031; minimum non-dominant side: *p* = 0.005; mean non-dominant side: *p* = 0.030), and at the hypothenar eminence (minimum dominant side: *p* = 0.025; minimum non-dominant side: *p* = 0.001; mean non-dominant side: *p* = 0.031) of the hands of women diagnosed with FM. On the other hand, for the control group, the intra-group analysis revealed a significant decrease in the temperature at the dorsal index fingertip minimum dominant (*p* = 0.031), at the palm thumb fingertip (maximum dominant side: *p* = 0.032; mean dominant side: *p* = 0.049), at the palm centre (maximum dominant side: *p* = 0.027; mean dominant side: *p* = 0.040), and at the thenar eminence (maximum dominant side: *p* = 0.03; maximum non-dominant side: *p* = 0.049; mean dominant side: *p* = 0.006). Figure 5 shows the differences in peripheral temperature of the dorsal and palmar surfaces of the hands following radiofrequency stressor application in a woman with FM.

## 4. Discussion

The results of this study show that after an RF intervention, FM patients presented a significant reduction in electrical threshold scores, electrical pain, and pressure pain thresholds compared to healthy women. With respect to peripheral hand temperatures, women with FM presented increased temperatures in the dorsal and palmar centres of the hands compared to the control group, while healthy women presented decreased temperatures in the thumb, index finger, and thenar eminence compared to the FM group. 

Studies in the scientific literature have linked an alteration of the autonomic nervous system (ANS) in FM patients to changes in thermal regulation [15]. Despite this, the alteration at ANS level affecting peripheral thermal regulation could also be the reason for decreased thresholds in response to a thermal heat stimulus.

Our results show reduced pain thresholds in the FM group after the application of radiofrequency. The elevated presence of substance P or CGRP in the blood reported in patients with FM [27] promotes neurogenic inflammation processes [8]; for this reason, applying radiofrequency, by generating an increased peripheral temperature in the FM group in an area in which the neurovascular response is altered at the base, could further promote these processes, thus generating the reduction in pain thresholds found in our results. In this sense, Hagiwara S. et al. [28], using a rat model, demonstrated the existence of serotonergic descending pathways that would inhibit the effects of pulsed radiofrequency on the function of the noradrenergic inhibitory system, thus generating a hyperalgesic reaction and not a reduction in pain [28]. These alterations at the nervous level, despite being found in rats, partially coincide with the results obtained by Albrecht et al. [5], in which noradrenergic function is also altered in women with FM. An involvement of these pathways could therefore be responsible for the decreased pain thresholds in FM patients. Given these data and the lack of previous publications on the subject, future studies are necessary to clarify these findings. 

On the other hand, AVAs play an important role in the body’s thermoregulation process, especially in the hand region [7]. The fact that the temperature recorded in the control group is lower after the application of radiofrequency could indicate that this microcirculation is functioning correctly compared to the FM group, as heat-induced vasoconstriction is generated, which would account for the decrease in these values. Although some authors have not found this mechanism to be present in humans [29,30,31], it is present in other mammals [32]. This lack of positive results may be due to the thermal stimulus used, as all of these studies employ similar methods [29,30,31]. The use of a deeper thermal stimulus, such as radiofrequency, could potentially trigger the activation of this mechanism. Otherwise, the increased temperature observed in the FM group may be due to the alteration of these structures at the nervous level [5,9,10], which could cause them to function incorrectly, thus preventing the heat-induced vasoconstriction effect from occurring and increasing or maintaining the vasodilation that would already be the baseline condition in women with FM [5]. These results would be in line with the presence of microvascular alterations and their relationship with the greater perception of pain observed in the group of FM sufferers compared to the control group. In this sense, de Tommaso M. et al. [33] pointed to the possible existence of a small-caliber nerve fiber pathology [33], especially C-fibres [34], which could affect the correct processing of thermal stimuli and contribute to increased pain perception and alterations in sensation [34], thus explaining some of the characteristic symptoms of FM, such as chronic fatigue [35].

### Study Limitations

This study has certain limitations. Firstly, due to the small sample size and the fact that it comprises a well-characterized population of female FM sufferers, our results may not be generalizable to other populations. Secondly, when measuring peripheral blood flow thermographically, there is intra-subject variability [36]; however, an acclimatization period was implemented [37]. Thirdly, the possibility of not having detected any neurological pathology in the participants or that these pathologies had not been previously diagnosed could affect the interpretation of the present results. Despite these limitations, this study has one particular strength. To our knowledge, this is the first study to attempt to establish a relationship between the vascular–thermal component and pain symptomatology in patients with FM after the application of radiofrequency. 

## 5. Conclusions

Women with FM present greater pain perception related to a decrease in pain thresholds, as well as an increase in peripheral temperature, after exposure to radiofrequency thermal stimulation. These findings reinforce the hypothesis that FM sufferers may have neurovascular disorders as well as possible alterations in thermogenesis processes related to pain perception. Future studies should therefore investigate the relationship between vascular function under different stressors and its association with pain to gain further knowledge to help in the diagnosis and treatment of this pathology.

## Figures and Tables

**Table 1:** Sociodemographic characteristics, comorbidities, and pharmacologic treatments in women with fibromyalgia and healthy women.

	Women with FM (n = 29)	Healthy Women (n = 17)	
Variable	Mean ± SD/Frequency (%)	Mean ± SD/Frequency (%)	*p*-Value
Age (years)	54.90 ± 7.52	58.12 ± 4.83	0.121
Height (cm)	159.24 ± 5.47	159.24 ± 7.28	0.997
Weight (kg)	68.66 ± 13.20	66.20 ± 11.80	0.530
BMI (kg/cm^2^)	28.29 ± 7.48	26.17 ± 4.69	0.298
Duration of FM (years)	15.44 ± 9.17	-	
Menopause status			
Pre-menopausal	21 (72.41)	14 (82.35)	0.446
Post-menopausal	8 (27.59)	3 (17.65)	
Smoking history			
Active smoker	14 (48.28)	10 (58.82)	
Non-smoker	15 (51.72)	7 (41.18)	
Comorbidity			
Chronic fatigue	10 (34.48)	0	0.001 *
Chemical hypersensitivity	3 (10.35)	0	
Painful bladder syndrome	7 (24.13)	2 (11.76)	
Non-comorbidity	9 (31.04)	15 (88.24)	
Pharmacologic history			
Corticosteroids	2 (6.90)	0	0.268
Analgesics	8 (27.59)	1 (5.88)	
Antidepressants	4 (13.80)	1 (5.88)	

* Significance level *p* < 0.05. Note: Data are expressed as mean ± standard deviation (SD) for quantitative variables and as frequency (%) for qualitative variables. Abbreviations: FM: fibromyalgia; BMI: body mass index.

## Data Availability

Data are available upon reasonable request. The datasets used and/or analyzed during the current study are available from the corresponding author upon reasonable request.

## Supplementary Materials

The following supporting information can be downloaded at: https://www.mdpi.com/article/10.3390/biomedicines12010142/s1. Table S1: Baseline, post-radiofrequency data, and the changes produced in each group (95% confidence interval) for pain intensity, electrical pain threshold, electrical pain intensity, and pressure pain threshold; Table S2: Baseline, post-radiofrequency data, and the changes produced in each group (95% confidence interval) for core body temperature, axillary temperature, and peripheral temperatures of palm sites of both hands; Table S3: Baseline, post-radiofrequency data, and the changes produced in each group (95% confidence interval) for peripheral temperatures of dorsal sites of both hands.
