# Supplementary material for: Vasodilatory Peripheral Response and Pain Levels following Radiofrequency Stressor Application in Women with Fibromyalgia
Source: Biomedicines. 2024 Jan 10;12(1):142. doi: 10.3390/biomedicines12010142 (PMC10813501; doi:10.3390/biomedicines12010142)
Supplement: Supplementary file 1 [file biomedicines-12-00142-s001.zip › biomedicines-2801537-supplementary.pdf]

Table S1. Baseline, post radiofrequency data, and the changes produced in each group (95% confidence interval) for pain intensity, electrical pain threshold, electrical pain intensity and pressure pain threshold.

	Baseline	Post-treatment (Post-radiofrequency)	Within-group Cohen's d	Score change	
				Within-group	Between-group
**VAS (mm)**					
Women with FM	72.07±18.78	66.17±26.45	0.26	5.89 (-3.351, 15.140)	11.43 (-3.354, 26.208)
Healthy control	12.94±17.23	18.47±21.94	0.28	-5.53 (-17.606, 6.542)	
**Pain threshold electric score (mA)**					
Women with FM	5.60±3.73	3.82±0.99	0.65	1.78 (0.010, 3.556)	2.51 (0.338, 4.679)*
Healthy control	4.90±1.57	5.63±1.67	0.45	-0.72 (-1.978, 0.527)	
**Pain electric score (mA)**					
Women with FM	11.68±8.60	5.03±1.29	1.08	6.65 (2.870, 10.428)	5.84 (1.272, 10.418)*
Healthy control	10.86±6.39	10.06±5.29	0.14	0.80 (-1.887, 3.495)	
**Pressure pain thresholds (kPa)**					
**Occiput**					
Women with FM					
D	0.73±0.47	0.90±0.57	0.32	-0.17 (-0.433, 0.084)	-1.75 (-2.368, -1.142)*
ND	0.74±0.58	0.90±0.53	0.29	-0.16 (-0.437, 0.118)	-1.16 (-1.810, -0.516)*
Healthy control					
D	3.51±1.20	1.93±0.86	1.51	1.58 (0.877, 2.284)	
ND	3.09±0.99	2.09±1.28	0.87	1.00 (0.265, 1.742)	
**Trapezius**					
Women with FM					
D	0.75±0.50	0.88±0.66	0.22	-0.13 (-0.347, 0.087)	-0.87 (-1.642, -0.104)*
ND	0.75±0.47	0.87±0.55	0.23	-0.12 (-0.303, 0.055)	-0.85 (-1.650, -0.057)*
Healthy control					
D	3.39±1.51	2.65±1.20	0.54	0.74 (-0.233, 1.719)	
ND	3.36±1.49	2.63±1.45	0.50	0.73 (-0.308, 1.767)	

Table S1. *Cont*.

	Baseline	Post-treatment (Post-radiofrequency)	Within-group Cohen's d	Score change	
				Within-group	Between-group
**Zygapophyseal joint**					
Women with FM					
D	1.01±0.71	0.84±0.55	0.27	0.17 (-0.087, 0.428)	-0.90 (-1.696, -0.103)
ND	0.93±0.66	0.87±0.68	0.09	0.06 (-0.221, 0.339)	-0.96 (-1.870, -0.044)
Healthy control					
D	3.21±1.85	2.14±0.96	0.73	1.07 (0.082, 2.058)	
ND	3.33±2.12	2.32±1.24	0.58	1.02 (-0.128, 2.160)	
**Supraspinatus**					
Women with FM					
D	1.04±0.77	0.76±0.49	0.43	0.28 (0.042, 0.524)	-0.37 (-1.355, 0.607)
ND	1.08±0.66	0.76±0.49	0.55	0.32 (0.035, 0.601)	-0.56 (-1.510, 0.390)
Healthy control					
D	3.82±2.02	3.16±1.89	0.34	0.66 (-0.629, 1.944)	
ND	3.93±1.83	3.05±1.67	0.50	0.88 (-0.318, 2.074)	
**Second rib**					
Women with FM					
D	0.75±0.46	0.65±0.38	0.24	0.10 (-0.078, 0.278)	-0.40 (-0.873, 0.081)
ND	0.78±0.56	0.59±0.32	0.42	0.19 (-0.014, 0.390)	-0.46 (-1.010, 0.092)
Healthy control					
D	2.48±1.16	1.98±1.10	0.44	0.50 (-0.075, 1.068)	
ND	2.52±1.00	1.87±0.90	0.68	0.65 (-0.016, 1.310)	
**Epicondyle**					
Women with FM					
D	0.79±0.52	0.78±0.53	0.02	0.01 (-0.217, 0.240)	-1.18 (-1.895, -0.475)*
ND	0.76±0.49	0.65±0.42	0.24	0.11 (-0.114, 0.344)	-1.21 (-1.946, -0.468)
Healthy control					
D	3.56±1.36	2.36±1.27	0.91	1.20 (0.316, 2.078)	
ND	3.82±1.60	2.49±1.44	0.87	1.32 (0.399, 2.246)	

Table S1. *Cont*.

	Baseline	Post-treatment (Post-radiofrequency)	Within-group Cohen's d	Score change	
				Within-group	Between-group
**Gluteus**					
Women with FM					
D	1.42±0.94	1.90±1.46	0.39	-0.48 (-0.864, -0.095)	-1.48 (-3.077, 0.114)*
ND	1.57±0.98	1.90±1.31	0.28	-0.33 (-0.727, 0.075)	-0.92 (-2.407, 0.570)*
Healthy control					
D	6.78±2.74	5.78±2.75	0.36	1.00 (-1.063, 3.067)	
ND	6.37±2.26	5.77±3.05	0.22	0.59 (-1.309, 2.495)	
**Greater trochanter**					
Women with FM					
D	1.86±1.09	1.63±0.93	0.23	0.23 (-0.122, 0.586)	-0.74 (-2.094, 0.612)
ND	1.93±1.09	1.52±0.84	0.42	0.41 (-0.030, 0.853)	-0.70 (-1.928, 0.522)
Healthy control					
D	6.13±2.51	5.16±2.23	0.41	0.97 (-0.763, 2.709)	
ND	6.12±2.29	5.00±1.97	0.52	1.11 (-0.367, 2.596)	
**Knee**					
Women with FM					
D	1.28±0.98	1.18±0.82	0.11	0.10 (-0.313, 0.518)	-1.97 (-3.158, -0.779)
ND	1.49±0.83	1.26±1.13	0.23	0.23 (-0.224, 0.694)	-1.51 (-2.828, -0.194)*
Healthy control					
D	6.51±2.47	4.44±2.25	0.88	2.07 (0.621, 3.521)	
ND	6.24±2.31	4.50±2.24	0.76	0.,75 (0.140, 3.352)	
**Second metacarpal**					
Women with FM					
D	0.97±0.52	0.99±0.70	0.03	-0.02 (-0.303, 0.261)	-1.26 (-1.975, -0.553)
ND	0.93±0.49	0.84±0.59	0.17	0.09 (-0.198, 0.371)	-1.17 (-1.839, -0.496)*
Healthy control					
D	3.69±1.30	2.44±1.39	0.93	1.24 (0.407, 2.078)	
ND	3.50±1.25	2.24±1.22	1.02	1.25 (0.484, 2.025)	

Table S1. *Cont*.

	Baseline	Post-treatment (Post-radiofrequency)	Within-group Cohen's d	Score change	
				Within-group	Between-group
**Anterior tibial**					
Women with FM					
D	1.37±0.99	1.39±0.85	0.02	-0.02 (-0.407, 0.359)	-0.71 (-1.828, 0.411)*
ND	1.61±1.10	1.14±0.69	0.51	0.47 (0.046, 0.893)	-0.67 (-1.927, 0.577)*
Healthy control					
D	5.30±2.29	4.61±2.07	0.32	0.68 (-0.687, 2.056)	
ND	5.43±2.19	4.29±2.14	0.53	1.14 (-0.393, 2.682)	

* Significant group × time interaction (analysis of variance, $P < 0.05$).

Note. Values are expressed as means ± standard deviation (SD) for baseline and post radiofrequency application, and as means (95% confidence interval) for within-group and between-group score changes. Abbreviations. VAS: visual analog pain scale; FM: Fibromyalgia; mA: milliamps; kPa: kilopascal; D: dominant; ND: non-dominant.

Table S2. Baseline, post radiofrequency data, and the changes produced in each group (95% confidence interval) for core body temperature, axillary temperature and peripheral temperatures of palm sites of both hands.

	Baseline	Post-treatment (Post-radiofrequency)	Within-group Cohen's d	Score change	
				Within-group	Between-group
**Tympanic temperature (ºC)**					
Women with FM	35.86±0.71	36.08±0.51	0.36	-0.22 (-0.486, 0.045)	0.78 (-1.232, -0.334)*
Healthy control	35.87±0.58	35.31±0.49	1.04	0.56 (0.166, 0.959)	
**Axillary temperature (ºC)**					
Women with FM	35.46±0.73	35.51±0.90	0.06	-0.52 (-0.442, 0.338)	0.89 (-1.585, -0.204)*
Healthy control	35.58±0.71	34.74±0.76	1.14	0.84 (0.205, 1.480)	
**Palmar sites of both hands**					
**Thumb fingertip maximum (ºC)**					
Women with FM					
D	32.05±3.12	32.35±3.10	0.10	-0.30 (-1.823, 1.223)	-2.12 (-4.425, 0.187)
ND	31.83±2.97	32.17±3.02	0.11	-0.34 (1-839, 1.153)	-1.72 (-4.129, 0.683)*
Healthy control					
D	30.18±3.31	28.36±3.45	0.54	1.82 (0.181, 3.457)	
ND	30.11±3.83	28.73±3.20	0.39	1.38 (-0.528, 3.288)	
**Thumb fingertip minimum (ºC)**					
Women with FM					
D	31.05±3.06	31.70±3.09	0.21	-0.66 (-2.147, 0.833)	-2.08 (-4.385, 0.221)
ND	30.72±2.97	31.56±3.01	0.28	-0.84 (-2.357, 0.686)	-1.52 (-3.988, 0.957)
Healthy control					
D	28.90±3.32	27.47±3.40	0.43	1.42 (-0.305, 3.155)	
ND	28.53±3.21	27.85±3.15	0.21	0.68 (-1.331, 2,691)	
**Thumb fingertip average (ºC)**					
Women with FM					
D	31.66±3.07	32.05±3.08	0.13	-0.39 (-1.865, 1.079)	-2.05 (-4.305, 0.206)
ND	31.43±2.96	31.89±3.04	0.15	-0.46 (-1.949, 1.034)	-1.78 (-4.175, 0.620)
Healthy control					
D	29.57±3.29	27.91±3.43	0.49	1.66 (-0.001, 3.314)	
ND	29.65±3.71	28.33±3.24	0.38	1.32 (-0.581,3.221)	

Table S2. *Cont*.

	Baseline	Post-treatment (Post-radiofrequency)	Within-group Cohen's d	Score change	
				Within-group	Between-group
**Index fingertip maximum (ºC)**					
Women with FM					
D	31.69±3.27	32.30±3.10	0.19	-0.61 (-2.162, 0.941)	-2.40 (-4.811, 0.002)*
ND	31.35±3.18	31.78±3.33	0.13	-0.43 (-2.075, 1.218)	-1.70 (-4.248, 0.841)
Healthy control					
D	29.55±3.61	27.76±3.58	0.50	1.79 (-0.030, 3.618)	
ND	29.10±3.99	27.82±3.50	0.34	1.27 (-0.636, 3.186)	
**Index fingertip minimum (ºC)**					
Women with FM					
D	30.86±3.12	31.56±3.15	0.22	-0.70 (-2.231, 0.824)	-2.44 (-4.797, -0.085)*
ND	30.09±3.06	28.30±3.85	0.51	-1.02 (-2.590, 0.540)	-2.46 (-4.889, -0.023)*
Healthy control					
D	28.61±3.54	26.87±3.44	0.50	1.74 (-0.023, 3.498)	
ND	28.30±3.85	26.87±3.26	0.40	1.43 (-0.423, 3.286)	
**Index fingertip average (ºC)**					
Women with FM					
D	32.10±3.29	32.53±3.11	0.13	-0.43 (-2.130, 1.266)	-2.39 (-4.762, -0.020)*
ND	30.87±3.15	31.43±3.34	0.17	-0.56 (-2.190, 1.076)	-1.88 (-4.400, 0.648)
Healthy control					
D	30.12±3.59	28.62±3.64	0.41	1.49 (-0.151, 3.140)	
ND	28.69±3.96	27.37±3.42	0.36	1.32 (-0.578, 3.216)	
**Middle fingertip maximum (ºC)**					
Women with FM					
D	31.33±3.29	31.88±3.23	0.17	-0.55 (-2.084, 0.976)	-2.05 (-4.458, 0.356)
ND	31.28±3.31	31.65±3.32	0.11	-0.36 (-2.044, 1.315)	-2.02 (-4.609, 0.568)
Healthy control					
D	28.79±3.85	27.29±3.49	0.41	1.50 (-0.379, 3.379)	
ND	29.03±3.99	27.37±3.59	0.44	1.66 (-0.275, 3.587)	

Table S2. *Cont*.

	Baseline	Post-treatment (Post-radiofrequency)	Within-group Cohen's d	Score change	
				Within-group	Between-group
**Middle fingertip minimum (ºC)**					
Women with FM					
D	30.41±3.16	31.19±3.29	0.24	-0.78 (-2.283, 0.726)	1.17 (-4.458, 0.264)
ND	30.33±3.18	30.92±3.40	0.18	-0.59 (-2.225, 1.046)	-1.81 (-4.318, 0.690)
Healthy control					
D	27.84±3.65	26.51±3.38	0.38	1.32 (-0.521, 3.158)	
ND	27.91±3.85	26.68±3.54	0.33	1.22 (-0.611, 3.061)	
**Middle fingertip average (ºC)**					
Women with FM					
D	30.86±3.24	31.51±3.27	0.20	-0.65 (-2.176, 0.869)	-2.03 (-4.415, 0.358)
ND	30.86±3.25	31.25±3.36	0.12	-0.39 (-2.061, 1.275)	-1.83 (-4.396, 0.735)
Healthy control					
D	28.30±3.74	26.92±3.46	0.38	1.37 (-0.480, 3.230)	
ND	28.45±3.93	27.01±3.57	0.38	1.44 (-0.468, 3.342)	
**Ring fingertip maximum (ºC)**					
Women with FM					
D	31.44±3.32	31.80±3.44	0.11	-0.37 (-1.963, 1.227)	-1.61 (-4.130, 0.907)
ND	31.51±3.53	31.62±3.57	0.03	-0.11 (-1.944, 1.723)	-1.50 (-4.299, 1.302)
Healthy control					
D	28.76±3.95	27.51±3.72	0.33	1.24 (-0.747, 3.235)	
ND	28.97±4.29	27.59±3.64	0.35	1.39 (-0.652, 3.427)	
**Ring fingertip minimum (ºC)**					
Women with FM					
D	30.42±3.27	31.06±3.37	0.19	-0.64 (-2.204, 0.918)	-1.71 (-4.159, 0.736)
ND	30.32±3.39	30.71±3.51	0.11	-0.39 (-2.116, 1.330)	-1.33 (-3.976, 1.316)
Healthy control					
D	27.74±3.84	26.67±3.51	0.29	1.07 (-0.834, 2.972)	
ND	27.57±4.06	26.64±3.38	0.25	0.94 (-1.019, 2.894)	

Table S2. *Cont*.

	Baseline	Post-treatment (Post-radiofrequency)	Within-group Cohen's d	Score change	
				Within-group	Between-group
**Ring fingertip average (ºC)**					
Women with FM					
D	30.93±3.30	31.42±3.40	0.15	-0.49 (-2.070, 1.084)	-1.60 (-4.091, 0.893)
ND	30.84±3.49	31.15±3.52	0.09	-0.31 (-2.089, 1.468)	-1.45 (-4.169, 1.273)
Healthy control					
D	28.22±3.91	27.12±3.64	0.29	1.11 (-0.866, 3.079)	
ND	28.24±4.12	27.10±3.55	0.30	1.14 (-0.853, 3.128)	
**Pinkie fingertip maximum (ºC)**					
Women with FM					
D	31.54±3.59	31.79±3.66	0.07	-0.25 (-1.993, 1.485)	-1.55 (-4.226, 1.131)
ND	31.57±3.54	31.53±3.84	0.01	0.04 (-1.857, 1.935)	-1.26 (-4.095, 1.574)
Healthy control					
D	28.66±4.01	27.37±3.81	0.33	1.29 (-0.702, 3.290)	
ND	28.72±4.16	27.41±3.64	0.33	1.30 (-0.637, 3.237)	
**Pinkie fingertip minimum (ºC)**					
Women with FM					
D	30.20±3.34	31.06±3.65	0.25	0.86 (-2.541, 0.820)	-2.06 (-4.649, 0.528)
ND	30.24±3.38	30.68±3.82	0.12	-0.44 (-2.209, 1.338)	-1.34 (-3.994, 1.310)
Healthy control					
D	27.68±3.85	26.48±3.56	0.32	1.20 (-0.727, 3.127)	
ND	27.46±3.95	26.55±3.38	0.25	0.91 (-0.905, 2.717)	
**Pinkie fingertip average (ºC)**					
Women with FM					
D	30.97±3.55	31.41±3.66	0.12	-0.44 (-2.149, 1.278)	-1.62 (-4.244, 1.010)
ND	30.97±3.43	31.06±3.82	0.02	-0.08 (-1.913, 1.749)	-1.08 (-3.834, 1.669)
Healthy control					
D	28.08±3.91	26.90±3.67	0.31	1.18 (-0.752, 3.114)	
ND	27.99±4.06	26.99±3.56	0.26	1.00 (-0.912, 2.912)	

Table S2. *Cont*.

	Baseline	Post-treatment (Post-radiofrequency)	Within-group Cohen's d	Score change	
				Within-group	Between-group
**Palm centre maximum(ºC)**					
Women with FM					
D	34.18±1.32	34.58±1.27	0.31	-0.40 (-1.061, 0.261)	-1.41 (-2.471, -0.342)*
ND	33.90±1.52	34.52±1.26	0.44	-0.62 (-1.356, 0.106)	-1.24 (-2.355, -0.133)*
Healthy control					
D	33.27±1.61	32.27±2.15	0.53	1.01 (0.132, 1.881)	
ND	32.81±1.75	32.19±2.21	0.31	0.62 (-0.178, 1.415)	
**Palm centre minimum (ºC)**					
Women with FM					
D	32.95±1.32	33.69±1.32	0.56	-0.73 (-1.390, -0.074)	-1.46 (-2.495, -0.419)*
ND	32.50±1.56	33.55±1.34	0.72	-1.06 (-1.775, -0.339)	-1.72 (-2.895, -0.544)*
Healthy control					
D	31.61±1.70	30.88±2.05	0.39	0.72 (-0.092, 1.542)	
ND	31.26±1.82	30.60±2.10	0.34	0.66 (-0.334, 1.659)	
**Palm centre average (ºC)**					
Women with FM					
D	33.65±1.29	34.15±1.30	0.39	-0.50 (-1.137, 0.137)	-1.37 (-2.395, -0.355)*
ND	33.32±1.50	34.11±1.29	0.56	-0.79 (-1.487, -0.084)	-1.41 (-2.493, -0.328)*
Healthy control					
D	32.58±1.56	31.71±2.06	0.48	0.87 (0.046, 1.704)	
ND	32.22±1.71	31.59±2.12	0.33	0.62 (-0.185, 1.435)	
**Thenar eminence maximum (ºC)**					
Women with FM					
D	33.84±1.30	34.12±1.51	0.20	-0.28 (-1.003, 0.439)	-1.99 (-3.191, -0.798)*
ND	33.52±1.62	34.03±1.53	0.32	-0.51 (-1.288, 0.259)	-1.67 (-2.973, -0.368)*
Healthy control					
D	33.06±1.50	31.35±2.36	0.86	1.71 (0.673, 2.752)	
ND	32.46±1.80	31.30±2.32	0.56	1.16 (-0.001, 2.313)	

Table S2. *Cont*.

	Baseline	Post-treatment (Post-radiofrequency)	Within-group Cohen's d	Score change	
				Within-group	Between-group
**Thenar eminence minimum (ºC)**					
Women with FM					
D	31.35±1.51	31.97±1.95	0.35	-0.62 (-1.479, 0.237)	-1.67 (-3.047, -0.296)*
ND	31.22±1.67	31.75±2.00	0.29	-0.53 (-1.481, 0.417)	-1.56 (-2.044, -0.071)*
Healthy control					
D	29.84±1.56	28.79±2.52	0.50	1.05 (-0.072, 2.172)	
ND	29.67±1.61	28.65±2.53	0.48	1.02 (-0.128, 2.178)	
**Thenar eminence average (ºC)**					
Women with FM					
D	32.50±1.32	32.89±1.81	0.25	-0.39 (-1.191, 0.406)	-1.85 (-3.110, -0.601)*
ND	32.18±1.53	32.88±1.79	0.42	-0.70 (-1.553, 0.153)	-1.80 (-3.171,-0.429)*
Healthy control					
D	31.21±1.51	29.75±2.45	0.72	1.46 (0.482, 2.443)	
ND	30.83±1.68	29.73±2.47	0.52	1.10 (-0.022, 2.222)	
**Hypothenar eminence maximum (ºC)**					
Women with FM					
D	33.82±1.52	34.19±1.52	0.24	-0.37 (-1.189, 0.454)	-0.86 (-2.151, 0.428)
ND	33.49±1.63	34.22±1.57	0.46	-0.73 (-1.619, 0.154)	-0.79 (-2.122, 0.546)
Healthy control					
D	32.64±1.48	32.14±2.37	0.25	0.49 (-0.511, 1.499)	
ND	32.31±1.68	32.26±2.55	0.02	0.06 (-0.874, 0.986)	
**Hypothenar eminence minimum (ºC)**					
Women with FM					
D	30.89±1.94	31.89±1.51	0.57	-1.00 (-1.871, -0.136)	-0.72 (-2.011, 0.579)
ND	30.12±2.23	32.12±1.61	0.02	-1.99 (-3.025, -0,960)	-0.87 (-2.494, 0.758)
Healthy control					
D	28.87±1.78	29.16±2.30	0.14	-0.29 (-1,168, 0.593)	
ND	28.12±2.08	29.24±2.28	0.51	-1.12 (-2.403, 0.153)	

Table S2. *Cont*.

	Baseline	Post-treatment (Post-radiofrequency)	Within-group Cohen's d	Score change	
				Within-group	Between-group
**Hypothenar eminence average (ºC)**					
Women with FM					
D	32.30±1.77	32.83±1.60	0.31	-0.53 (-1.399, 0.335)	-0.96 (-2.229, 0.315)
ND	32.00±1.85	33.00±1.59	0.58	-1.00 (-1.911, -0.096)	-1.07 (-2.417, 0.272)
Healthy control					
D	30.64±1.71	30.21±2.20	0.22	0.42 (-0.387, 1.237)	
ND	30.49±1.85	30.42±2.35	0.03	0.07 (-0.823, 0.961)	

*Significant group × time interaction (analysis of variance, $P < 0.05$).

Note. Values are expressed as means ± standard deviation (SD) for baseline and post radiofrequency application, and as means (95% confidence interval) for within-group and between-group score changes. Abbreviations. ºC: Celsius degree; FM: Fibromyalgia; D: dominant; ND: non-dominant.

Table S3. Baseline, post radiofrequency data, and the changes produced in each group (95% confidence interval) for peripheral temperatures of dorsal sites of both hands.

	Baseline	Post-treatment (Post-radiofrequency)	Within-group Cohen's d	Score change	
				Within-group	Between-group
**Dorsal sites of both hands**					
**Thumb fingertip maximum (ºC)**					
Women with FM					
D	33.32±3.28	33.62±2.92	0.10	-0.30 (-1.883, 1.290)	-1.74 (-4.084, 0.608)
ND	33.19±3.11	33.30±3.04	0.04	-0.11 (-1.751, 1.537)	-1.40 (-3.863, 1.061)
Healthy control					
D	31.58±3.48	30.13±3.75	0.40	1.44 (-0.202, 3.085)	
ND	31.22±3.53	29.93±3.67	0.36	1.29 (-0.490, 3.079)	
**Thumb fingertip minimum (ºC)**					
Women with FM					
D	31.73±3.00	32.36±2.98	0.21	-0.63 (-2.165, 0,908)	-1.92 (-4.182, 0.336)
ND	31.84±2.91	32.21±3.01	0.12	-0.37 (-1.971, 1.228)	-1.81 (-4.192 ,0.579)
Healthy control					
D	29.69±3.45	28.39±3.42	0.38	1.29 (-0.264, 2.852)	
ND	29.58±3.43	28.14±3.31	0.43	1.43 (-0.275, 3.145)	
**Thumb fingertip average (ºC)**					
Women with FM					
D	32.73±3.18	33.06±2.95	0.11	-0.33 (-1.906, 1.249)	-1.68 (-4.015, 0.652)
ND	32.63±3.04	32.79±3.02	0.05	-0.15 (-1.778, 1.478)	-1.56 (-3.986, 0.867)
Healthy control					
D	30.73±3.50	29.38±3.64	0.38	1.35 (-0.284, 2.990)	
ND	30.51±3.51	29.10±3.48	0.40	1.41 (-0.342, 3.154)	

Table S3. *Cont*.

	Baseline	Post-treatment (Post-radiofrequency)	Within-group Cohen's d	Score change	
				Within-group	Between-group
**Index fingertip maximum (ºC)**					
Women with FM					
D	32.57±3.36	32.95±3.15	0.12	-0.38 (-2.097, 1.340)	-1.76 (-4.280, 0.758)
ND	32.26±3.30	32.59±3.29	0.10	-0.32 (-2.071, 1.428)	-1.39 (-3.971, 1.187)
Healthy control					
D	30.65±3.57	29.27±3.73	0.38	1.38 (-0.339, 3.104)	
ND	30.19±3.89	29.12±3.60	0.28	1.07 (-0.720, 2.861)	
**Index fingertip minimum (ºC)**					
Women with FM					
D	31.36±3.15	32.01±3.11	0.21	-0.65 (-2.306, 1.013)	-2.35 (-4.736, 0.032)
ND	31.17±3.09	31.82±3.28	0.20	-0.65 (-2.354, 1.054)	-1.99 (-4.477, 0.483)
Healthy control					
D	29.32±3.56	27.62±3.35	0.49	1.71 (0.179, 3.233)	
ND	28.81±3.70	27.46±3.18	0.39	1.35 (-0.311, 3.005)	
**Index fingertip average (ºC)**					
Women with FM					
D	32.10±3.29	32.53±3.11	0.13	-0.43 (-2.130, 1.266)	-1.93 (-4.395, 0.543)
ND	31.84±3.22	32.22±3.28	0.12	-0.39 (-2.121, 1.343)	-1.57 (-4.108, 0.976)
Healthy control					
D	30.11±3.59	28.62±3.64	0.41	1.49 (-0.152, 3.140)	
ND	29.65±3.83	28.48±3.44	0.32	1.18 (-0.567, 2.920)	
**Middle fingertip maximum (ºC)**					
Women with FM					
D	32.36±3.44	32.69±3.33	0.10	-0.32 (-2.037, 1.394)	-1.65 (-4.182, 0.881)
ND	32.26±3.35	32.41±3.29	0.04	-0.15 (-1.937, 1.644)	-1.13 (-3.735, 1.465)
Healthy control					
D	30.42±3.65	29.09±3.74	0.36	1.33 (-0.433, 3.092)	
ND	30.06±3.64	29.07±3.65	0.27	0.99 (-0.738, 2.714)	

Table S3. *Cont*.

	Baseline	Post-treatment (Post-radiofrequency)	Within-group Cohen's d	Score change	
				Within-group	Between-group
**Middle fingertip minimum (ºC)**					
Women with FM					
D	31.24±3.27	31.83±3.32	0.18	-0.59 (-2.262, 1.076)	-2.07 (-4.474, 0.335)
ND	31.16±3.25	31.70±3.36	0.16	-0.54 (-2.310, 1.232)	-1.88 (-4.418, 0.657)
Healthy control					
D	29.01±3.43	27.53±3.36	0.43	1.48 (-0.078, 3.031)	
ND	28.88±3.70	27.54±3.37	0.38	1.34 (-0.267, 2.950)	
**Middle fingertip average (ºC)**					
Women with FM					
D	31.94±3.38	32.27±3.29	0.10	-0.34 (-2.043, 1.371)	-1.74 (-4.221, 0.750)
ND	31.90±3.33	32.07±3.32	0.05	-0.18 (-1.96, 1.608)	-1.30 (-3.875, 1.282)
Healthy control					
D	29.86±3.60	28.46±3.60	0.39	1.40 (-0.265, 3.065)	
ND	29.58±3.66	28.46±3.59	0.31	1.12 (-0.560, 2.795)	
**Ring fingertip maximum (ºC)**					
Women with FM					
D	32.61±3.53	32.81±3.47	0.06	-0.20 (-1.993, 1.593)	-1.43 (-4.117, 1.258)
ND	32.20±3.55	32.34±3.49	0.04	-1.14 (-2.067, 1.788)	-0.93 (-3.721, 1.854)
Healthy control					
D	30.23±3.84	29.00±3.87	0.32	1.23 (-0.723, 3.182)	
ND	29.84±3.86	29.05±3.70	0.21	0.79 (-1.031, 2.619)	
**Ring fingertip minimum (ºC)**					
Women with FM					
D	31.39±3.47	31.84±3.48	0.13	-0.45 (-2.254, 1.354)	-1.96 (-4.561, 0.649)
ND	31.05±3.48	31.47±3.46	0.12	-0.42 (-2.341, 1.505)	-1.55 (-4.281, 1.175)
Healthy control					
D	28.81±3.59	27.30±3.48	0.43	1.51 (-0.193, 3.205)	
ND	28.51±3.88	27.38±3.39	0.31	1.13 (-0.534, 2.804)	

Table S3. *Cont*.

	Baseline	Post-treatment (Post-radiofrequency)	Within-group Cohen's d	Score change	
				Within-group	Between-group
**Ring fingertip average (ºC)**					
Women with FM					
D	32.10±3.48	32.30±3.43	0.06	-0.20 (-1.992, 1.585)	-1.51 (-4.161, 1.142)
ND	31.79±3.50	31.92±3.48	0.04	-0.13 (-2.049, 1.785)	-1.06 (-3.807, 1.684)
Healthy control					
D	29.61±3.75	28.30±3.74	0.35	1.31 (-0.563, 3.175)	
ND	29.29±3.85	28.36±3.56	0.25	0.93 (-0.809, 2.668)	
**Pinkie fingertip maximum (ºC)**					
Women with FM					
D	32.09±3.57	32.42±3.52	0.09	-0.33 (-2.172, 1.508)	-1.50 (-4.187, 1.193)
ND	32.04±3.46	32.06±3.70	0.01	-0.02 (-1.966, 1.923)	-0.73 (-3.522, 2.056)
Healthy control					
D	29.70±3.92	28.53±3.62	0.31	1.16 (-0.660, 2.989)	
ND	29.48±3.91	28.77±3.48	0.19	0.71 (-1.065, 2.489)	
**Pinkie fingertip minimum (ºC)**					
Women with FM					
D	30.64±3.37	31.54±3.55	0.26	-0.90 (-2.703, 0.910)	-2.38 (-4.965, 0.196)
ND	31.16±3.25	31.70±3.36	0.16	-0.54 (-2.310, 1.232)	-1.51 (-4.169, 1.153)
Healthy control					
D	28.39±3.68	26.91±3.33	0.42	1.49 (-0.130, 3.106)	
ND	28.88±3.70	27.54±3.37	0.38	1.34 (-0.267, 2.950)	
**Pinkie fingertip average (ºC)**					
Women with FM					
D	31.58±3.52	32.02±3.51	0.12	-0.44 (-2.272, 1.393)	-1.84 (-4.484, 0.806)
ND	31.54±3.40	31.68±3.73	0.04	-0.14 (-2.070, 1.784)	-0.94 (-3.695, 1.809)
Healthy control					
D	29.22±3.85	27.82±3.48	0.38	1.40 (-0.322, 3.122)	
ND	28.93±3.85	28.13±3.39	0.22	0.80 (-0.924, 2.524)	

Table S3. *Cont*.

	Baseline	Post-treatment (Post-radiofrequency)	Within-group Cohen's d	Score change	
				Within-group	Between-group
**Dorsal centre maximum(ºC)**					
Women with FM					
D	32.84±1.38	33.89±1.27	0.79	-1.04 (-1.684, -0.401)	-1.45 (-2.497, -0.400)*
ND	32.36±1.61	33.68±1.28	0.91	-1.32 (-2.059, -0.584)	-1.30 (-2.487, -0.120)*
Healthy control					
D	31.86±1.82	31.46±2.04	0.21	0.41 (-0.494, 1.306)	
ND	31.61±1.92	31.63±2.11	0.01	-0.18 (-1.005, 0.970)	
**Dorsal centre minimum (ºC)**					
Women with FM					
D	31.51±1.64	33.07±1.31	1.05	-1.56 (-2.288, -0.841)	-2.00 (-3.143, -0.868)*
ND	31.29±1.76	32.84±1.32	0.99	-1.56 (-2.309, -0.805)	-1.71 (-2.895, -0.525)*
Healthy control					
D	30.82±1.81	30.38±2.08	0.23	0.44 (-0.474, 1.356)	
ND	30.59±1.85	30.43±2.03	0.08	0.15 (-0.806, 1.112)	
**Dorsal centre average (ºC)**					
Women with FM					
D	32.25±1.50	33.46±1.29	0.86	-1.21 (-1.898, -0.516)	-1.64 (-2.749, -0.536)*
ND	31.85±1.70	33.25±1.28	0.93	-1.41 (-2.147, -0.668)	-1.50 (-2.661, -0.342)*
Healthy control					
D	31.37±1.79	30.93±2.09	0.23	0.43 (-0.484, 1.354)	
ND	31.12±1.88	31.02±2.03	0.05	0.09 (-0.834, 1.022)	

*Significant group × time interaction (analysis of variance, $P < 0.05$).

Note. Values are expressed as means ± standard deviation (SD) for baseline and post radiofrequency application, and as means (95% confidence interval) for within-group and between-group score changes. Abbreviations. ºC: Celsius degree; FM: Fibromyalgia; D: dominant; ND: non-dominant.
